# Ethnic inclusion in medicine: the ineffectiveness of the ‘Black, Asian and Minority Ethnic’ metric to measure progress

**DOI:** 10.3399/BJGPO.2020.0155

**Published:** 2020-11-25

**Authors:** Jason Kwasi Sarfo-Annin

**Affiliations:** 1 NIHR Academic Clinical Fellow in Primary Care, Centre for Academic Primary Care, University of Bristol, Bristol, UK

**Keywords:** Service organisation, Ethnic groups, Minority groups, Primary healthcare, General practice

The medical profession is a success story for ethnic diversity. Approximately 45% of hospital doctors and at least 32% of GPs in the United Kingdom are from minority groups.^1,2^ Fourteen per cent of the UK population is non-White, with a further 5.5% identifying as a minority White group.^3^ However, success is marred by problems of ‘inclusion’ of minority ethnic doctors.^4^ Inclusion is defined as *‘* *the achievement of a work environment in which all individuals are treated fairly and respectfully, have equal access to opportunities and resources, and can contribute fully to the* *organisation* *’s success.* *’* ^5^ The King’s Fund, a health think-tank, has recently highlighted that senior leadership and managerial positions are predominately held by White British and male persons.^6^ There has been longstanding discussion regarding inclusion in medicine on the basis of characteristics such as gender and socioeconomic status.^7,8^ However, the events of 2020 — such as the death of George Floyd, the *Black Lives Matter* protests, and the impact of COVID-19 on ethnic minority groups — have led to a focus on race and ethnicity.

The use of a ‘Rooney Rule’, a form of affirmative action to improve racial diversity and/or inclusion, has been touted as a solution to a lack of ethnic inclusion in British medicine. This rule was created in 2003 by the National Football League (NFL) — where at least one candidate from a minority ethnic group must be interviewed by an American football team for the head coach position.^9^ Affirmative action is a quick way to reduce — or attempt to reduce — racial inequality and exclusion. That does not necessarily make it the best or the only way. In 2003, when the Rooney Rule was introduced by the NFL, there were three Black head coaches.^10^ As of August 2020, there are three Black head coaches. The NFL has since amended the rule, implicitly acknowledging the rule’s failure to achieve the outcome desired.^11^ Affirmative action will likely fail in the long run if there is a failure to engage with the hard and complex work of making the structural and cultural changes required to improve racial inclusion.^10^

Whether accurate or not, there is a concern that the introduction of affirmative action will lead to a group of sub-prime minority ethnic doctors in leadership roles. For many people, the journey matters just as much as the destination. Some, given the choice, would prefer to become a millionaire by building and owning a business rather than winning a lottery jackpot. Similarly, there are minority ethnic doctors who want to be seen as having reached a position of authority as a result of their skills and personal qualities, and not primarily because of a protected characteristic. Advocates of affirmative action need to bear this in mind.

A major problem with affirmative action for minority ethnic doctors in the UK is that the widely used term ‘BAME’ (Black, Asian and Minority Ethnic) is presumed to be the best metric to use. It has a dreadful utility in general, and in British medicine particular. Doctors of South Asian heritage represent two-thirds of the BAME population.^1,2^ This size confers influence and power. Concerns regarding racial bias in the Clinical Skills Assessment, the examination that GP Registrars in the UK undertake as part of their training, affected doctors from all minority ethnic groups. However, it was the British Association of Physicians of Indian Origin who took the Royal College of General Practitioners (RCGP) to court.^12,13^

For a ‘Rooney Rule’ to achieve representative diversity, roughly 35–40% of leadership positions should be held by people of minority ethnic backgrounds. Approximately one in every three of these representatives *shouldn’t* be of South Asian heritage. Affirmative action on the basis of inclusion in the BAME definition would treat smaller minority ethnic groups equally to the largest. Therefore a situation could arise where there are greater numbers of minority ethnic peoples in leadership roles, but they are overwhelmingly of South Asian heritage. There are already many examples of South Asian doctors in leadership roles: the current Chair of the British Medical Association (BMA), who is also the immediate past chair of the General Practice Committee; the current NHS Medical Director of Primary Care and the immediate past-President of the RCGP and of the Royal College of Paediatrics. Looking at medical leadership though the lens of someone with Chinese heritage, there have been few individuals with Chinese heritage who have reached posts of national standing. Similarly, through an Afrocentric lens, there has never been a Black President or Chair of a Royal Medical College, nor Chair of the BMA. Doctors of Chinese heritage are outnumbered by an order of twelve by South Asian doctors, and Black doctors are outnumbered by an order of six.^1,2^ Differences in representation between various minority groups in senior positions is to be expected, but need not be potentially exacerbated by affirmative action. As BAME is defined widely as a term for non-White ethnic groups, it is often overlooked that a BAME ‘Rooney Rule’ excludes minority groups such as White Europeans, and those from Gypsy or Traveller backgrounds.

Where do we go from here to improve inclusion? The first step is to collect and use much more granular data on ethnicity. The ethnicity data provided by the General Medical Council on GPs is poor without a full breakdown of ethnicity categories.^2^ The use of the UK census ethnicity groups should be the minimum standard for any organisation in health care.^14^ There are justifiable reasons for census categories such as ‘Black African’ to be further subdivided to ‘Black West African’, ‘East African’, and so on. The term ‘Asian’ or ‘South Asian’ is also not granular enough a descriptive term. It is well known that educational attainment in UK schools of Pakistani and Bangladeshi pupils is below that of Indians.^15,16^ It is important to track the progress of these groups independently in health care, as entry to medical school is based on educational attainment at school. Most importantly, while there are some cultural overlaps between some different ethnic groups, the worldview and experiences of a Black Caribbean person will not be the same as that of a Chinese person. It is crucial that people recognise and remember the diversity within and between minority ethnic groups.

The next step is to devise strategies to make sure that talented people from minority ethnic groups are noticed and given the opportunities they deserve. Tools to hold organisations to account on their progress and targets are critical. It is key that minority ethnic groups are engaged in defining these targets and the accountability process. Factors that impact on inclusion but are poorly defined and/or difficult to measure will be fundamental to improvements. However, I believe that this ‘grey area’ — which is overlooked in favour of measurable targets — is what requires the hardest work. This will require numerous different approaches throughout organisations.^17^ Within this, there are two areas that deserve specific mention: mentorship and engagement.

The phenomenon where mentors (and mentees) are inclined to voluntarily engage in a mentoring relationship with those who remind them of themselves can be problematic. There must be some shared familiarity and ease for any mentoring relationship to work. That said, within medicine, there is a growing number of women and ethnic minority doctors, who have a small pool of mentors to choose from — if they are seeking people who reflect them. Both mentors at a senior level and mentees must be open to seeking out relationships with people of the opposite sex or of different ethnicity.

It is very important for individuals and organisations to keep engaged and to continuously talk to each other. This provides the opportunity for people to express and learn from nuances that are rarely spoken about. Creating a culture where people from minority ethnic groups can discuss topics that are very difficult, or perhaps others wouldn't have previously been interested in hearing, takes time. How to go about building in mechanisms to make sure these voices are heard will require tailoring to the organisational and local needs. Criticism should be expected and embraced by organisations. Yet, activities performed by organisations must clearly evidence the learning from listening exercises and criticisms. Accountability to minority ethnic groups is essential to avoid accusations of paying corporate lip service.

BAME is far too broad a term to use as a metric of ethnic inclusion or affirmative action as there is heterogeneity within and between minority ethnic groups. Refined data on the ethnicity of doctors is required to picture and improve awareness of this, alongside open discussion and organisational accountability of actions. These should be prerequisite steps before considering affirmative action, to avoid its failure to improve inclusion in the long term. Inclusion of ethnic minorities is a complex challenge which will need continuous efforts. Doing nothing favours the status quo and those who are currently more likely to obtain senior and management roles within health care.
